# Health-related quality of life, self-reported impairments and activities of daily living in relation to muscle function in post-polio syndrome

**DOI:** 10.1186/s41687-020-00226-5

**Published:** 2020-07-16

**Authors:** Vanya Gocheva, Patricia Hafner, Anna-Lena Orsini, Simone Schmidt, Sabine Schaedelin, Nicole Rueedi, Daniela Rubino-Nacht, Peter Weber, Dirk Fischer

**Affiliations:** 1 Division of Neuropediatrics and Developmental Medicine, University Children’s Hospital of Basel (UKBB),University of Basel, Spitalstrasse 33, Postfach, 4056 Basel, Switzerland; 2 Division of Neurology, University Hospital Basel, Basel, Switzerland; 3 Department of Neurology, General Hospital Hietzing with Neurological Center Rosenhügel, Vienna, Austria; 4 Department of Clinical Research, Clinical Trial Unit, University Hospital Basel, Basel, Switzerland

**Keywords:** Post-polio syndrome, Health-related quality of life, Impairments, Activities of daily living, Patient-reported outcomes, Motor function

## Abstract

**Background:**

The symptoms of post-polio syndrome (PPS) and its resulting disabilities can affect quality of life and the ability to perform daily activities. No study has comprehensively analysed how various patient-reported outcome measures (PROMs) are associated with objectively assessed physical function in patients with PPS.

**Aim:**

To investigate health-related quality of life (HRQOL), self-reported impairments and activities of daily living during 6 months and evaluate their association with clinical muscle function outcomes in individuals with PPS.

**Methods:**

Twenty-seven patients with PPS were included in the study. At baseline and 6 months, patients were administered PROMs measuring HRQOL (WHOQOL-BREF), self-reported impairments related to PPS (SIPP-RS) and activities of daily living (IBM-FRS). Clinical muscle function outcomes included 6 min walking distance (6MWD) and motor function measure (MFM).

**Results:**

There were no changes in self-reported impairments (25.52 to 24.93, *p* = 0.40), activities of daily living (33.89 to 33.30, *p* = 0.20), 6MWD (391.52 to 401.85, *p* = 0.30) and MFM (83.87 to 85.46, *p* = 0.14) during 6 months, while the HRQOL psychological health decreased during this period (76.85 to 72.38, *p* = 0.05). A strong association was found between activities of daily living and clinical muscle function outcomes (6MWD: *ß* = 0.02, 95% CI: 0.02;0.03, *t* = 6.88, *p* < 0.01; MFM: *ß* = 0.25, 95% CI: 0.17;0.33, *t* = 6.69, *p* < 0.01). Self-reported impairments and HRQOL domains were not associated with the clinical muscle outcomes.

**Conclusions:**

Study findings indicate that objectively measured walking and motor abilities do not reflect patient’s perspectives of their HRQOL and impairment due to PPS. More research is needed to assess changes over time and capture clinically meaningful changes in individuals with PPS and to increase the understanding of how the patient’s perspective of disability measured by PROMs is related to objectively measured walking and motor abilities.

**Trial registration:**

ClinicalTrials.gov Identifier (NCT02801071) registered June 15, 2016.

## Background

The post-polio syndrome (PPS) is a condition that affects polio survivors years after an acute poliomyelitis infections leading to flaccid paralysis. Survivors often (partially) recover from these paralysis [1]. PPS is defined as “the development of new muscle weakness and fatigue in skeletal or bulbar muscles, unrelated to any known cause, beginning 25-30 years after an acute attack of paralytic poliomyelitis” [2]. Additional symptoms of PPS include muscle atrophy, generalized fatigue, muscle, and joint pain and sensitivity to cold [3]. Primary symptoms and impairments such as sleep disturbances, memory and concentration difficulties may be disabling in certain areas of life and may affect independence [4–6]. While studies report that 40% to 80% of polio survivors already have PPS, the actual incidence of PPS is still unknown [7]. To date, the exact cause of PPS is still unclear. The most widely accepted hypothesis refers the symptoms to a distal degeneration of axons in the greatly enlarged motor units developed during recovery from acute paralytic poliomyelitis [8]. As no curative treatment is available for PPS, rehabilitation management is considered the mainstay of treatment [9].

The symptoms of PPS and the resulting disabilities may affect quality of life. Previous studies reported poorer health-related quality of life (HRQOL) in persons with PPS compared to the general population [4, 10]. In addition, the limitations caused by PPS often influence the ability to perform daily activities and lead to a wide range of problems in daily life such as difficulties in dressing, toileting, transfer etc. [11–13]. Moreover, the majority of patients with PPS report especially dependence in the most mobility-related activities such as cleaning, shopping and transportation [14].

In clinical practice, objective measurements of muscle function and walking distance are commonly used when the consequences of PPS are evaluated [15, 16]. However, objective measures of functional outcome only partially capture the different aspects of impairments and walking limitations that persons with PPS perceive. The patient’s perspective should be taken into consideration for a more comprehensive understanding of the disease’s impact. Patient-reported outcome measures (PROMs) are increasingly advocated and used to achieve this [17]. PROMs are used extensively in a clinical research setting, the Food and Drug Administration has recognized their importance in natural history and clinical trials [18]. PROMs allow patients to consider their real-world experiences integrated over time and provide a broader and deeper understanding of persons’ own perception of everyday difficulties [19].

To date, little has been reported on if and how different PROMs relate to objectively assessed physical function and its changes over time [20, 21]. Therefore, a more comprehensive analysis is warranted of how patient-reported variables such as self-reported impairments related to PPS, HRQOL and activities of daily living are associated to motor function. Self-reported impairments refers to impairments that are commonly reported by patients with PPS and are indirectly related to their prior experience of polio such as muscle weakness, muscle fatigue, breathing difficulties. To the best of our knowledge, no study has investigated these associations.

The aim of this study was therefore to assess self-reported evaluations with three PROMs regarding HRQOL, impairments related to PPS and activities of daily living during 6 months in patients with PPS. Additionally, we aimed to examine whether there was an association between the PROMs and objectively assessed muscle function and walking distance.

## Methods

### Study design

This study is a prospective observational study involving patients with PPS recruited at the Division of Neuropediatrics, University Children’s Hospital Basel in Switzerland and followed for 6 months. This study is part of a lager randomised controlled trial designed to assess the efficacy of L-citrulline in patients with PPS, which involves a 24-week observational (untreated) natural history period followed by a 24-week treatment period. This analysis used baseline and 6 months data of the untreated participants with PPS during the natural history period. Details of the clinical trial protocol and design are reported elsewhere [22].

### Study population

For this study, participants were recruited among the PPS patient organization in Switzerland (www.polio.ch). Eligible participants were included in the study only after providing written informed consent. Ethics approval had been obtained from the local Ethics Committee (EKNZ 2015–221) and the National Swiss Drug Agency (Swissmedic, Reference number: 2016DR2067). The study was registered at ClinicalTrials.gov (NCT02801071) prior to starting recruitment. The PROMs and the clinical measures investigated in this study were all part of the larger randomised controlled study and therefore part of the original informed consent. Inclusion criteria for the larger study were defined as follows:

i. prior paralytic poliomyelitis with evidence of lower motor neuron loss
ii. a period of partial or complete functional recovery after acute paralytic poliomyelitis
iii. slowly progressive and persistent new muscle weakness or decreased endurance, with or without generalized fatigue, muscle atrophy, or muscle and joint pain
iv. ≥18 years of age at inclusion
v. ability to walk at least 150 m in the 6 min walking distance test with or without walking stick(s), and
vi. no other significant medical condition or malignancy.

Out of thirty-three screened participants with PPS two patients were excluded from participation because they did not meet inclusion criteria. Between baseline and follow-up assessment at 6 months four patients withdrew informed consent, resulting in a final number of 27 participants.

### Measurements

#### Self-reported impairments in persons with late effects of polio rating scale

The Self-Reported Impairments in Persons with Late Effects of Polio Rating Scale (SIPP-RS) is a 13-item self-report assessment of impairments related to PPS [23]. The participants rate how much they have been bothered directly (i.e., muscle weakness, fatigue) or indirectly (i.e., sensory disturbances, mood swings) by various impairments related to late effects of polio during the past 2 weeks. The items consider: muscle weakness, muscle fatigue, muscle and/or joint pain during physical activity and at rest, sensory disturbance, breathing difficulties during physical activity and at rest, cold intolerance, general fatigue, sleep disturbances, concentration difficulties, memory difficulties, and mood swings. Response categories range from 1 (not at all) to 4 (extremely). The total sum score ranges from 13 to 52 points, a higher score indicating more self-reported impairments. The SIPP-RS has been Rasch analyzed and is unidimensional [23] and it has also been examined for test-retest reliability with an intraclass correlation coefficient of 0.88 [24], but content validity has not been demonstrated so far.

#### Inclusion body myositis functional rating scale

The Inclusion Body Myositis Functional Rating Scale (IBM-FRS) is a 10-item functional rating scale that assesses activities of daily living [25]. Respondents rate their functional ability at current time in 10 areas including swallowing, handwriting, use of utensils, fine motor tasks, dressing, hygiene, turning in bed, standing, walking and climbing stairs. Response categories range from 4 being normal to 0 being unable to perform. The total score ranges from 40 (best functional status) to 0 (complete dependency). The IBM-FRS has been shown to be a reliable and valid measure of disease severity in inclusion body myositis [25, 26]. The IBM-FRS is known to be a sensitive measure of disorders affecting the peripheral motor nerves or muscles in inclusion body myositis [25]. Therefore, and due to the clinical similarities of inclusion body myositis and PPS (late adult onset, slowly progressive weakness and atrophy, asymmetric affection of proximal and distal limb muscles, and lack of central nervous system involvement) [2, 3], the IBM-FRS was used to assess activities of daily living in this trial.

#### World Health Organisation quality of life abbreviated scale

The World Health Organisation Quality of Life Abbreviated Scale (WHOQOL-BREF) is a 26-item scale that assesses an individual’s HRQOL [27]. Response categories range from 1 to 5, with higher scores indicating a better HRQOL. The WHOQOL-BREF was scored after its administration to the study participants; the raw scores were converted to transformed scores. The first transformation converts scores to a range of 4–20 and the second transformation converts domain scores to a 0–100 scale. The domain scores show good content validity, discriminant validity and internal consistency [28]. This questionnaire assesses HRQOL over the past 2 weeks.

#### 6 Minute walking distance

In medical literature, numerous timed clinical functional assessments have been reported to assess to monitor the disease progression. The 6 min walking distance (6MWD) test is one of the most popular clinical tests used for assessment of muscle function and fatigue in patients with neuromuscular disorders [29, 30]. It is a validated tool to measure the distance that an individual is able to walk over a total of 6 min on a hard, flat surface. The aim of the test is to walk as far as possible in 6 min.

#### Motor function measure

The motor function measure (MFM) is a validated quantitative scale used for assessment of motor abilities of both ambulant and non-ambulant patients with neuromuscular disorders [31]. It includes 32 items that evaluate three dimensions of motor performance at current time, including specific motor functions, such as transfers and standing posture, proximal and axial motor function, and distal motor function. Each item is scored on a scale from 0 (does not initiate movement) to 3 (completes the item with a standard pattern). The items are scored and summed to comprise a total score involving all of the motor dimensions, where the maximum represents normal motor function (100%). In this study, the MFM total score was analysed. Each participant was assessed by a trained physiotherapist individually in a room containing a mat, a stretcher and the material required to answer the scale.

### Procedure

All consecutive patients attending the study centre who fulfilled the inclusion criteria were enrolled in the study. As we wished to obtain the best compliance in the functional assessments, the PROMs were filled in after the functional tasks. Data were collected at baseline and at 6 months follow-up assessment.

### Data analysis

Descriptive statistics were calculated for the continuous variables of mean, standard deviation and for the categorical variables of frequencies and percentages. Normality of the endpoints was assessed using the normal probability plot (QQ-plot). One sample t-tests were performed to compare the HRQOL scores of patients with PPS to data from general German population [32]. Paired t-tests were used to assess the change over time between baseline and 6 months follow-up visit of the PROMs and functional measures. In addition, the association between PROMs and functional data were assessed using linear mixed effects models. The outcome variables were the WHOQOL-BREF domain scores, the SIPP-RS total value and IBM-FRS total value. The MFM and 6MWD were included at the corresponding visit as additional covariates. The visit number was included as a fixed effect and participants as random effect. The coefficient estimates (*ß*) is reported together with 95% confidence intervals, the *t* and *p* values. Statistical analyses were performed using R, version 3.4.4.

## Results

### Characteristics of participants with PPS

A total of 27 participants with PPS (mean age = 65.48 years, SD = 4.80) had both baseline and follow-up data and were included in the analysis. Participants included 15 males (56%) and 12 females (44%). Regarding marital status at study start, 19 participants were married (70%), 3 were divorced (11%), 3 patients were single (11%), 1 was separated (4%) and 1 was widowed (4%). The highest education with greater representation was secondary school (*n* = 12, 44%), followed by university degree (*n* = 8, 30%), high school (*n* = 4, 15%), and PhD (*n* = 3, 11%).

### Baseline data

A summary of baseline PROM scores and functional data are shown in Table 1. All clinical tests were performed safely without any major fall during the assessment.

**Table 1:** Summary of PROMs and clinical outcomes at baseline (*n* = 27)

	Mean (±SD)	Possible range
**PROMs**		
WHOQOL-BREF		
Physical health	72.09 (±14.99)	0–100
Psychological health	76.85 (±16.11)	0–100
Social relationships	78.70 (±13.54)	0–100
Environmental health	84.14 (±10.79)	0–100
SIPP-RS	25.52 (±5.07)	13–52
IBM-FRS	33.89 (±3.75)	0–40
**Clinical outcomes**		
6MWD	391.52 (±132.24)	
MFM	83.87 (±12.85)	0–100

### Self-reported impairments related to PPS

The mean score of the self-reported impairments was 25.52 (SD 5.07) out of 52 points and the median 26.00. The most frequent impairments (rated as ‘quite a bit’ or ‘extremely’) that the participants reported were: for example muscle fatigue (18 participants, 67%), muscle weakness (15 participants, 56%), muscle and/or joint pain during physical activity (7 participants, 26%), breathing difficulties during physical activity (7 participants, 26%), and sleep disturbances (7 participants, 26%).

### Activities of daily living

The mean score of the activities of daily living was 33.89 (SD 3.75) out of 40 and the median 35.00. Seven participants (26%) reported limitations (“being unable to perform” or “requires assistance”) in their ability to stand up from sitting position independently. Over 15% (4 participants) reported limitations in their ability to climb stairs and 3.7% (1 participant) reported limitations in their ability to walk.

### HRQOL in patients with PPS in comparison to normative data

**Fig. 1:**
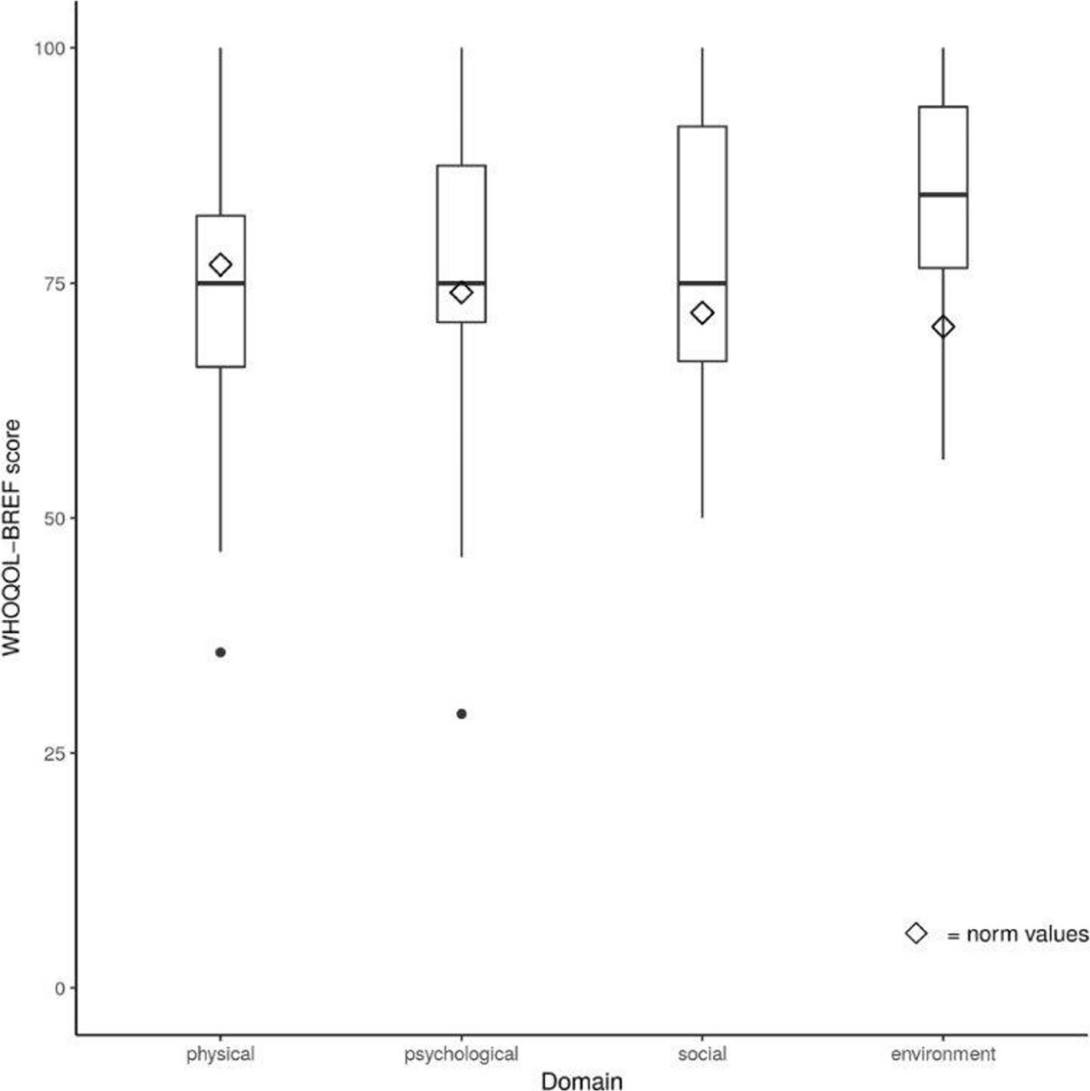
Distribution of the WHOQOL-BREF subscales at baseline of study participants compared to normative data

Table 2 shows the comparison of HRQOL between participants with PPS and normative data of general German population (*n* = 2073). Analysis revealed that participants with PPS reported significantly higher scores in the social relationships and the environmental health domains compared to general population (see Fig. 1). The physical and the psychological domains in PPS patients however did not significantly differ from the general population.

**Table 2:** Comparison of HRQOL scores among participants with PPS and healthy general population from normative data

	Participants with PPS *n* = 27	General population *n* = 2073	Difference (95% CI)	*t*	*p*
WHOQOL-BREF					
Physical health	72.09 (±14.99)	76.92 (±17.68)	−4.83 (− 10.76, 1.10)	− 1.67	0.11
Psychological health	76.85 (±16.11)	74.02 (±15.68)	2.83 (−3.54, 9.20)	0.91	0.37
Social relationships	78.70 (±13.54)	71.83 (±18.52)	6.87 (1.52, 12.23)	2.64	0.01*
Environmental health	84.14 (±10.79)	70.38 (±14.17)	13.76 (9.50, 18.03)	6.63	< 0.01*

Note: WHOQOL-BREF: A higher score indicates better health-related quality of life. *p* significant values in bold. *p ≤ 0.01 or above

### Longitudinal data

**Fig. 2:**
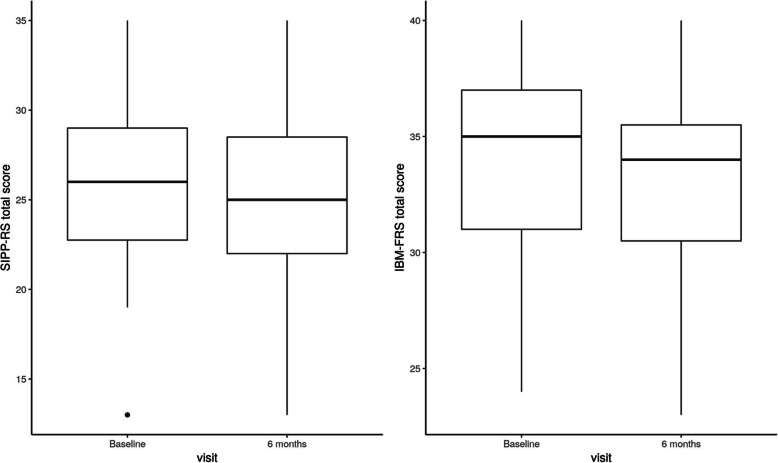
Change over time for the SIPP-RS and the IBM-FRS

Table 3 shows comparison between baseline and 6 months follow-up visit for the PROMs and functional data. The selected clinical outcome measures detected no change in physical function over the 6-months period (6MWD: 391.52 to 401.85, *p* = 0.30; MFM: 83.87 to 85.46, *p* = 0.14). The analysis of the HRQOL scores yielded a decrease between baseline and 6 months in the psychological domain (76.85 to 72.38, *p* = 0.05). No change over time could be found for the physical (72.09 to 69.86, *p* = 0.25), the social relationships (78.70 to 76.85, *p* = 0.35) and the environmental domains (84.14 to 82.06, *p* = 0.32). As shown in Fig. 2, no difference could be found between baseline and 6 months for patients’ self-reported impairments related to PPS (25.52 to 24.93, *p* = 0.40) and activities of daily living (33.89 to 33.30, *p* = 0.20).

**Table 3:** Comparison between baseline and 6 months follow-up visit for PROMs and clinical outcomes

	Baseline Mean (±SD)	6 months Mean (±SD)	Difference between the means (95% CI)	*t*	*p*
**PROMs**					
WHOQOL-BREF					
Physical health	72.09 (±14.99)	69.86 (±16.29)	−2.23 (−6.14, 1.68)	− 1.17	0.25
Psychological health	76.85 (±16.11)	72.38 (±17.42)	−4.48 (− 8.86, − 0.09)	− 2.10	0.05*
Social relationships	78.70 (±13.54)	76.85 (±13.54)	−1.85 (− 5.87, 2.17)	− 0.95	0.35
Environmental health	84.14 (±10.79)	82.06 (±12.24)	−2.08 (−6.28, 2.12)	−1.02	0.32
SIPP-RS	25.52 (±5.07)	24.93 (±5.35)	−0.59 (−2.02, 0.83)	− 0.86	0.40
IBM-FRS	33.89 (±3.75)	33.30 (±4.58)	−0.59 (−1.53, 0.34)	−1.31	0.20
**Clinical outcomes**					
6MWD	391.52 (±132.24)	401.85 (±148.10)	10.33 (−9.96, 30.63)	1.05	0.30
MFM	83.87 (±12.85)	85.46 (±12.19)	1.58 (−0.58, 3.74)	1.50	0.14

Note: WHOQOL-BREF: A higher score indicates better health-related quality of life. SIPP-RS: A higher score indicates more self-reported impairments. IBM-FRS: A higher score indicates better functional status. 6MWD: A higher score indicates a longer distance in 6 min. MFM: A higher score indicates better motor function

*p* significant values in bold. *p ≤ 0.05

### Association between PROMs and functional outcome measures

**Fig. 3:**
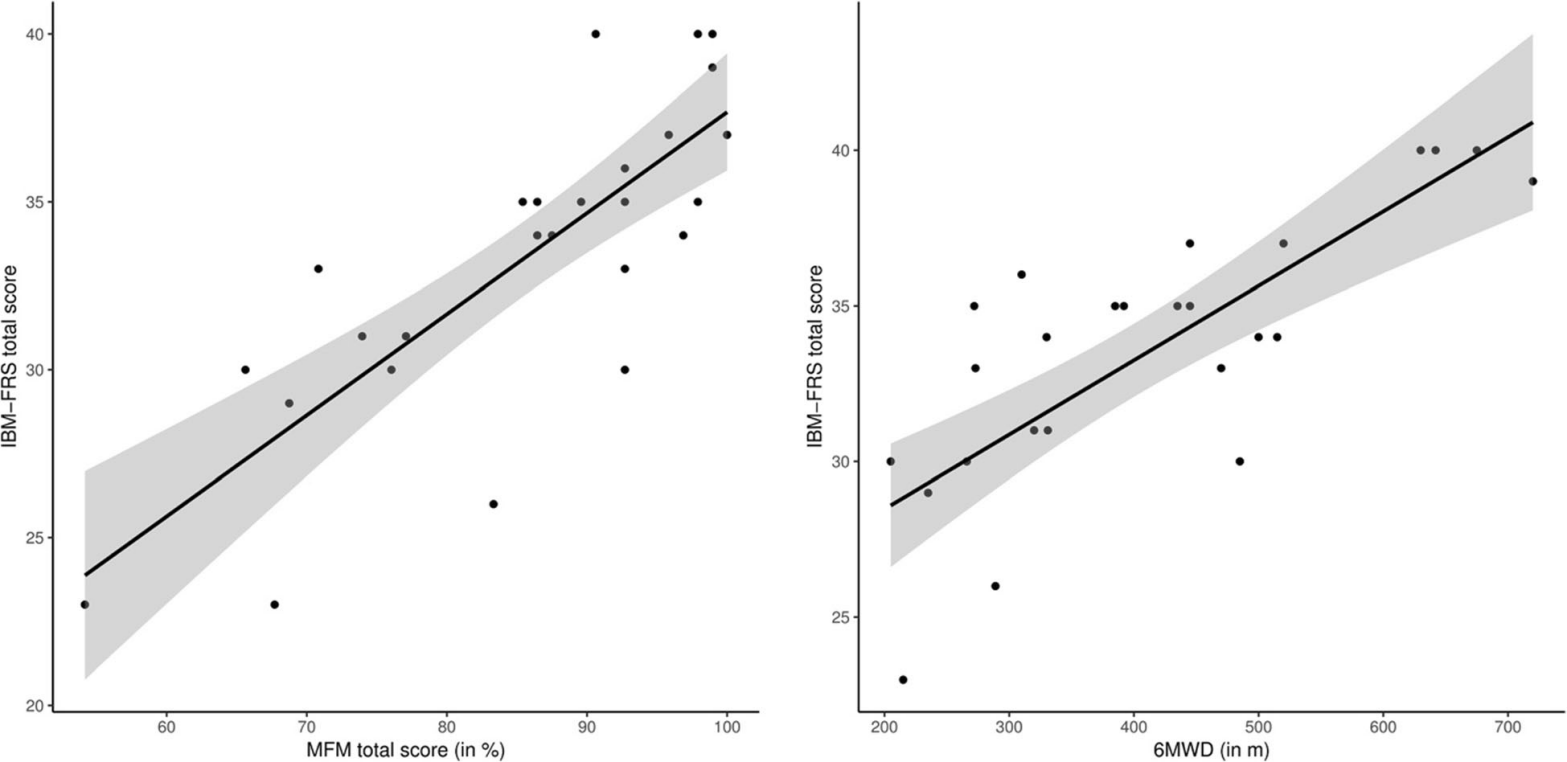
The relationship between the IBM-FRS and the clinical outcomes at baseline. The grey surface represents the 95% CI

Linear mixed model analysis revealed a strong association between the IBM-FRS and the 6MWD (*ß* = 0.02, 95% CI: 0.02;0.03, *t* = 6.88, *p* < 0.01), indicating that participants with PPS who were able to walk a further distance in 6 min experienced fewer limitations in activities in daily living. Moreover, a strong association was found between the IBM-FRS and the MFM (*ß* = 0.25, 95% CI: 0.17;0.33, *t* = 6.69, *p* < 0.01), demonstrating that patients with PPS with poorer motor function experience greater impairment on activities of daily living. The association of the IBM-FRS and the clinical outcome measures at baseline is presented in Fig. 3.

Analysis revealed no association between the WHOQOL-BREF physical domain and the 6MWD (*ß* = 0.02, 95% CI: − 0.02;0.05, *t* = 1.00, *p* = 0.33) and the MFM (*ß* = 0.04, 95% CI: − 0.36;0.42, *t* = 0.18, *p* = 0.86). Further, the psychological domain was not associated with the 6MWD (*ß* = 0.01, 95% CI: − 0.03;0.05, *t* = 0.56, *p* = 0.58) and the MFM (*ß* = − 0.06, 95% CI: − 0.48;0.37, *t* = − 0.28, *p* = 0.78). The same pattern was detected for the association between the clinical outcomes and the social domain (6MWD: *ß* = 0.01, 95% CI: − 0.02;0.04, *t* = 0.76, *p* = 0.45; MFM: *ß* = 0.23, 95% CI: − 0.09;0.55, *t* = 1.44, *p* = 0.16) and the environmental domain (6MWD: *ß* = 0.01, 95% CI: − 0.01;0.04, *t* = 1.15, *p* = 0.26; MFM (*ß* = 0.17, 95% CI: − 0.11;0.47, *t* = 1.21, *p* = 0.23).

Both the 6MWD and the MFM were not associated with the SIPP-RS (6MWD: *ß* = − 0.01, 95% CI: − 0.02;0.00, *t* = − 1.35, *p* = 0.19; MFM: *ß* = − 0.04, 95% CI: − 0.17;0.08, *t* = − 0.68, *p* = 0.50).

## Discussion

The PPS is a condition that leads to a life-long disability, with a variety of impairments that can increase over time and affect a person’s motor function, walking ability and quality of life.

Interestingly, participants reported better average HRQOL scores of social relationships and environmental health scores compared to general population, a finding that has not been reported before. A possible explanation could be that the majority of our participants live with a partner and receive regularly help and support. Several studies reported that social support is important for people who have contracted a disease [33, 34]. Social support, patients’ ability to manage stressors, as well as their ability to adjust to disability may minimize the importance of physical ability and therefore play an important role in maintaining mental health [35, 36]. Another possible explanation might be the relative low number of patients included in our study, thereby overestimating positive findings of individual patients. It is important to note that the possible explanations being discussed for this finding are hypotheses and therefore need to be tested in future studies.

Furthermore, our results revealed that patients with PPS reported comparable average HRQOL scores of physical and psychological health compared to healthy adults. Regarding the psychological health, Jacob and Shapira reported in their study normal emotional functioning in patients with PPS which is in line with our finding [12] .Previous reports on patients with PPS suggest that physical limitations are the major contributing factor to the impaired HRQOL [4, 12, 20, 21, 37], therefore our result is inconsistent with previous literature which may be due to our small sample size. The most common measures used in the literature to assess HRQOL were the Nottingham Health Profile [38] and the Short Form Health Survey [39]. They do share some overlap with the WHOQOL-BREF, however the physical functioning domains differ in content to the physical health of the WHOQOL-BREF as they measure the concept closest to functional capacity (including items such as lifting or carrying groceries; reaching for things; climbing stairs; bending over; walking more than a mile; bathing or dressing self; standing for long). The domain physical health of the WHOQOL-BREF incorporates the following facets: dependence on medicinal substances and medical aids, energy and fatigue, mobility, pain and discomfort, sleep and rest, work capacity, and activities of daily living. This suggests that the different questionnaires measure different aspects of physical activities. Another explanation could be that patients with PPS had been living with the effects of polio for many years, thus they had learnt to live with the changes caused by the disease. Coping strategies were developed and employed so they felt that they had a “good life” and their physical impairments did not affect their HRQOL [11, 36].

In this study, the participants were on average moderately affected by their impairments. The most often reported impairments (muscle fatigue, muscle weakness, muscle and/or joint pain during physical activity) are exemplary for people with PPS, therefore this finding is consistent with results of recent studies [16, 40]. Few participants reported breathing difficulties during physical activity and sleep disturbances, impairments that are more common in the recent literature [41], which demonstrates that the degree of self-reported impairments in persons with PPS can vary considerably. Further, the most common self-reported limitations were the ability to stand up from sitting position independently, the ability to climb stairs and limitations in their ability to walk. These impairments of daily life are in agreement with previous studies measured by other self-reported instruments [16, 40, 42] and emphasize the importance to assess several aspects of walking, not only walking distance and motor function.

Limited PROMs and physical function data are available on disease progression of PPS. The majority of studies so far used cross-sectional research designs measuring HRQOL at a single time point [4, 12, 20, 37]. In our study a decrease after the 6 months observation was only found for the HRQOL psychological health scores. The authors have no information regarding intervening events such as death of a spouse or forced retirement which could have precipitated a decline in psychological health. No changes of self-reported impairments related to PPS and activities of daily living were found after 6 months follow-up. Neither an objective disease progression was found in the MFM or 6MWD.

Still, as HRQOL psychological health was declining even in a short observational period HRQOL assessments may be used in clinical trials to obtain additional information on disease evolution compared to self-reported and objectively assessed physical disability. Further longer lasting natural history studies are recommended to get more objective data on different aspects of PPS disease progression.

Another interesting finding we observed is that the activities of daily living measured by the IBM-FRS are strongly associated with the 6MWD and the MFM. In accordance, the IBM-FRS was shown to correlate to traditional measures of efficacy in muscle testing in inclusion body myositis [25]. A closer look at the individual items possibly explains why the IBM-FRS correlated so well with clinical muscle function outcomes, while the PPS condition specific questionnaire SIPP-RS did not. The IBM-FRS mainly focuses on muscle groups essential to the activities of daily living, such as handgrip and quadriceps function [43], while the SIPP-RS reflects also secondary symptoms such as sleep disturbances, memory difficulties, and mood swings that may not parallel to functional changes.

To the best of our knowledge, this study is the first that assessed the association of various PROMs and several objective motor function measures and, therefore, our findings cannot be compared with some of the existing studies. The majority of previous studies in individuals with PPS have focused on the association between self-reported gait performance and a specific impairment. This study, in which several PROMs were used, adds new knowledge and increases our understanding of how a variety of self-reported impairments in persons with PPS can impact walking and motor abilities. However, more research is needed to increase understanding of how these self-reported impairments are related to objectively measured walking and motor abilities and to capture minimally important differences and clinically meaningful changes in individuals with PPS.

There are a number of important limitations of this analysis. A clear limitation of the study is the small sample size, which decreases the statistical power of the study. Sample size calculations for the study were based on the primary endpoint (6MWD) on the larger randomised controlled trial. A major limitation of this study was that one of the inclusion criteria was set to ensure that participants had a higher level of mobility (ability to walk 350 m in 6 min). Thus, this showed no major shift in health-related quality of life and motor function. In future studies, patients with broader range of function (also lower functioning patients) should be included. Although analysis corrected for baseline values was carried out, it is possible that important covariates such as fatigue, comorbidities etc. that may have had an impact were missed. Based on our data collected only from one site in Switzerland, the generalizability of our findings is reduced. Another limitation is the short observation period of 6 months. More research and long-term studies, including long-term follow-up visits (at least 1 year or more), are needed to establish if the observed trends are stable over longer periods.

## Conclusions

Self-reported impairments, HRQOL domains, activities of daily living and muscle function outcomes remained stable during 6 months in patients with PPS, except for the HRQOL psychological health domain which declined during this period. Clinical muscle outcomes had no statistically significant relationship with HRQOL and self-reported impairments of PPS patients while a significant relationship was observed with the activities of daily living. More research is needed to assess changes over time and capture clinically meaningful changes in individuals with PPS and to increase the understanding of how the patient’s perspective of disability measured by PROMs is related to objectively measured walking and motor abilities.

## Data Availability

Data used in the analysis is available upon request from the corresponding author. Patient-level data remains confidential under patient data privacy regulations.

## Abbreviations

6MWD: 6 min walking distance
HRQOL: Health-related quality of life
IBM-FRS: Inclusion body myositis – functional rating scale
MFM: Motor function measurement
PPS: Post-polio syndrome
PROM: Patient-reported outcome measure
SIPP-RS: Self-reported impairments in persons with late effects of polio rating scale
WHOQOL-BREF: World health organization (WHO) quality of life abbreviated scale
